# Switching on the Lights for Gene Therapy

**DOI:** 10.1371/journal.pone.0000528

**Published:** 2007-06-13

**Authors:** Alexandra Winkeler, Miguel Sena-Esteves, Leonie E.M. Paulis, Hongfeng Li, Yannic Waerzeggers, Benedikt Rückriem, Uwe Himmelreich, Markus Klein, Parisa Monfared, Maria A. Rueger, Michael Heneka, Stefan Vollmar, Mathias Hoehn, Cornel Fraefel, Rudolf Graf, Klaus Wienhard, Wolf D. Heiss, Andreas H. Jacobs

**Affiliations:** Laboratory for Gene Therapy and Molecular Imaging at the Max Planck-Institute for Neurological Research, Center for Molecular Medicine (CMMC) and Departments of Neurology and Radiology at the University of Cologne, Cologne, Germany; City of Hope Medical Center & Beckman Research Institue, United States of America

## Abstract

Strategies for non-invasive and quantitative imaging of gene expression *in vivo* have been developed over the past decade. Non-invasive assessment of the *dynamics* of gene regulation is of interest for the detection of endogenous disease-specific biological alterations (e.g., signal transduction) and for monitoring the induction and regulation of therapeutic genes (e.g., gene therapy). To demonstrate that non-invasive imaging of regulated expression of any type of gene after *in vivo* transduction by versatile vectors is feasible, we generated regulatable herpes simplex virus type 1 (HSV-1) amplicon vectors carrying hormone (mifepristone) or antibiotic (tetracycline) regulated promoters driving the proportional co-expression of two marker genes. Regulated gene expression was monitored by fluorescence microscopy in culture and by positron emission tomography (PET) or bioluminescence (BLI) *in vivo*. The induction levels evaluated in glioma models varied depending on the dose of inductor. With fluorescence microscopy and BLI being the tools for assessing gene expression in culture and animal models, and with PET being the technology for possible application in humans, the generated vectors may serve to non-invasively monitor the dynamics of any gene of interest which is proportionally co-expressed with the respective imaging marker gene in research applications aiming towards translation into clinical application.

## Introduction

Molecular imaging has become an important technology to non-invasively assess and monitor disease-specific biological processes *in vivo*. It is based on nuclear, magnetic resonance and optical imaging. In recent years reporter systems for these imaging techniques have been developed allowing the analysis of dynamic processes of endogenous and exogenous gene expression in living animals [1]–[4].

Moreover, imaging is being used to further develop new gene–and cell-based therapies [5], [6]. To non-invasively assess exogenous gene expression, e.g., imaging expression of a therapeutic transgene, strategies have been developed to couple and proportionally co-express a reporter gene with a transgene of interest [7]–[10]. The expression of the gene of interest is indirectly localized and quantified by the assessment of the marker gene. An ideal marker gene is not endogenously expressed in the host tissue and codes for an enzyme or receptor. Expression of the marker gene in transduced cells leads to enzyme-mediated trapping and accumulation of a specific substrate in the cell or by receptor-mediated specific binding on the cell surface. One of the most commonly used reporter genes in PET imaging is the HSV-1 thymidine kinase gene (HSV-1-*tk*). Saito [11] and Tjuvajev et al. [12] first described the HSV-1-*tk* as a suitable reporter gene to follow herpes virus infection and to image the expression of gene transduction *in vivo*.

Expression of reporter genes can be driven by different types of promoters or enhancer elements to monitor e.g., tissue-specific gene expression or transcriptional regulation [13]. Reporter systems bearing a reporter gene under control of endogenous enhancer elements have been used to monitor transcriptional regulation of p53-dependent genes [14], albumin gene expression [15] as well as T-cell activation [16].

Besides these endogenously regulated systems, inducible vector systems have been developed that allow the regulation of transduced genes in response to an exogenous compound. Various types of inducible systems have been established, regulated by e.g., antibiotics, hormones, heat shock or heavy metal ions [17]–[21]. One widely used regulated gene expression system is the tetracycline-responsive system developed by Gossen and Bujard [17] that is based on control elements of the tetracycline–resistance operon of *E. coli*. In this Tet-On/Off system a tetracycline derivative (doxycycline) binds to the reverse tetracycline-responsive transactivator (rtTA) and induces its binding to the Tet-controlled minimal CMV-promoter and, thus, its activation that leads to expression of the controlled gene [22]. A modification of this system uses a bi-directional doxycycline inducible promoter (P_BI-1_) controlling the expression of two independent genes [23]. Another regulatable system is the mifepristone or RU486 inducible system (“Gene-Switch”) [24]. The basis is an inducible chimeric transcription factor, consisting of (i) the ligand binding domain (LBD) of a mutant progesterone receptor that binds the progesterone antagonist RU 486 (mifepristone); (ii) the DNA binding domain from the yeast transcription factor Gal4 (GAL4DBD); and (iii) the activation domain from the HSV-1 protein VP16 or the human p65 [25]. Both, the Tet- and the Gene Switch system have been shown to be suitable tools to regulate gene expression *in vivo* [25]–[30] but rarely have been monitored by means of molecular imaging. Sun et al. were the first to image gene induction in a tumor xenograft model in living animals by using *ex vivo* transduced stable cell clones expressing the regulator and regulated genes [31].

Here, we report for the first time on the *quantitative dynamics* of regulated gene expression after transduction of co-regulated imaging genes by HSV-1 amplicon vectors containing doxycycline- or mifepristone -regulated elements [32]. The HSV-1 amplicon vectors include different reporter genes under control of the respective regulatory elements. Induced gene expression was quantified in cell culture (fluorescence microscopy) and *in vivo* (BLI, PET). Our universal HSV-1 amplicon vectors allow (i) efficient transduction of transgenes *in vivo*; (ii) induction of gene expression; and (iii) non-invasive assessment of the *dynamics* of the location and magnitude of any gene expression co-regulated with the marker gene *in vivo.*

## Results

### Construction of regulated HSV-1 amplicons expressing the PET-imaging gene HSV-*tk39* or HSV-1-*tkgfp17* as well as optical imaging genes

**Figure 1:**
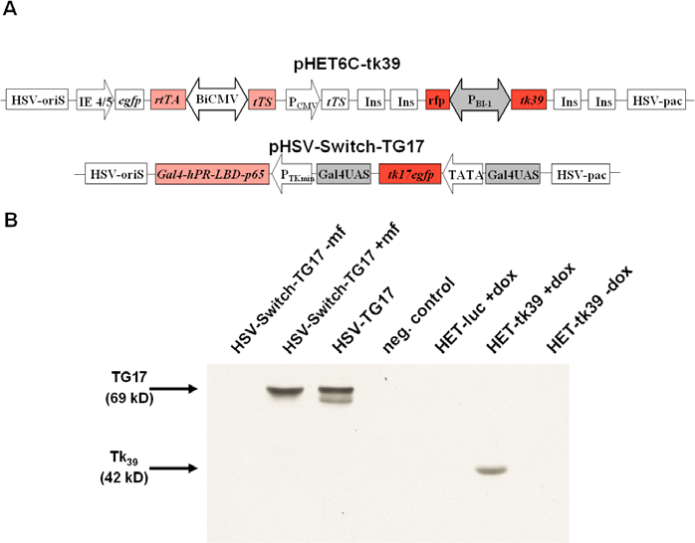
Inducible HSV-amplicon constructs. (A) The doxycycline-regulated HET6C-*tk39* vector consists of a bi-directional CMV promoter (BiCMV) controlling gene expression of the transactivator rtTA and the tetracycline controlled transcriptional silencer tTS [35]. A bi-directional tet-responsive promoter (P_BI-1_) controls expression of the marker genes *rfp* and *HSV-1-tk39*. In the absence of doxycycline, the silencer tTS binds to P_BI-1_, thus repressing background activity of this promoter. The presence of doxycycline leads to its release and binding of rtTA instead, thereby inducing gene expression of reporter genes *rfp* and *tk39.* The plasmid components of the mifepristone-inducible HSV-Switch-TG17 vector encode the chimeric transactivator *Gal4-hPR-LBD-p65* and the PET-reporter gene *HSV-1-tkgfp* under the control of a synthetic promoter consisting of four GAL4 upstream response elements and a TATA box. TG17 gene expression is induced by the chimeric transactivator protein in the presence of mifepristone. (B) Western Blot analysis of reporter expression was performed by infecting human Gli36ΔEGFR cells with HSV-Switch-TG17, HSV-TG17, HET6C-*tk39* or HET6C-*luc*, respectively. Protein signals for the fusion protein TG17 were only obtained by infection of cells with HSV-TG17, where expression of TG17 is under control of the constitutive CMV-promoter, or HSV-Switch-TG17 in the presence of the inducer mifepristone. The doublet band visible in HSV-TG17 infected cells may be due to sample processing with reducing agents and has already been seen and described for other TKGFP fusion constructs as well as wt HSV-thymidine kinase. TK39-protein signal was detectable only in cells that were infected with HET6C-*tk39* and treated with doxycycline.

Two different regulated HSV-1 amplicon vectors were constructed based on the Tet-system (HET6C-*tk39*) [17], [22] and on the antiprogestin system (HSV-Switch-TG17) [24] (**Figure 1A**). In HET6C-*tk39*, the gene for the specific HSV-1-*tk* mutant, *tk39* [33], as well as the *rfp* gene are under control of a bi-directional tetracycline-responsive promoter (P_BI-1_). In HSV-Switch-TG17, the *tkgfp17* fusion gene [34] was placed under transcriptional control of the Gal4 Upstream Activation Sequences (UAS). Thus, induction of gene expression can be detected in cell culture by fluorescence microscopy via RFP or eGFP expression and *in vivo* by PET via TK expression. For bioluminescence imaging the HET6C-*luc* vector was used, where *tk39* is substituted by the luciferase gene (*luc*). All HSV-1 amplicon vectors contain transactivator coding sequences and the regulated target genes; HET6C vectors also contain a tetracycline controlled transcriptional silencer tTS [35] (**Figure 1A**).

To verify whether expression of viral TK is regulated by the respective inducer (mifepristone or doxycycline), human Gli36ΔEGFR cells were infected with HET6C-*tk39* and HSV-Switch-TG17, respectively, and protein expression was assessed by Western Blot. The fusion protein TG17 was expressed only in cells infected with HSV-Switch-TG17 in the presence of mifepristone (**Figure 1B**, lane2). HSV-TG17 (CMV promoter) infected cells served as positive control (**Figure 1B**, lane 3). Likewise, expression of TK39 was only observed in HET6C-*tk39* infected cells after addition of doxycycline (**Figure 1B**, lane 6).

### Time- and dose-dependent induction of RFP and TKGFP expression in cell culture

**Figure 2:**
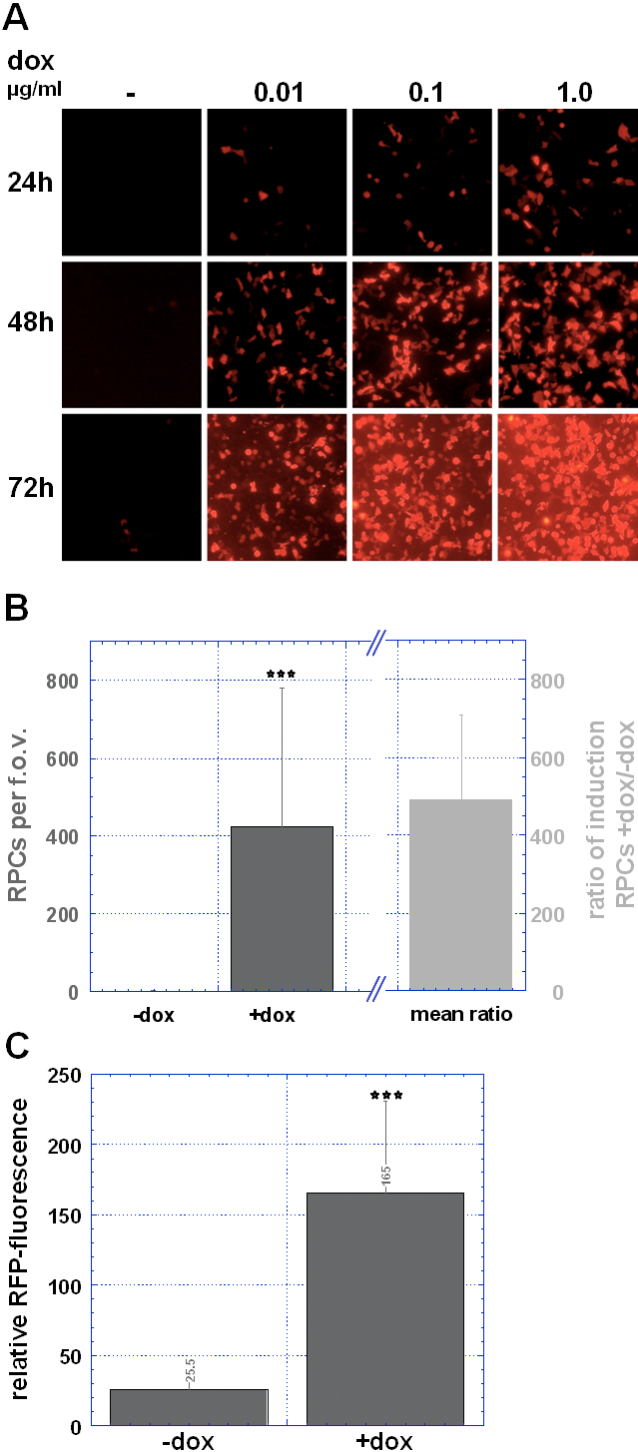
Doxycycline-induced RFP-expression. (A) RFP-expression in culture mediated by HET6C-*tk39*. Fluorescence microscopy of RFP-expressing cells that were infected with HET6C-*tk39* in the presence or absence of doxycycline. Although expression of RFP in HET6C-*tk39* infected but untreated cells is very tightly regulated, we find some leakiness of the construct that is indicated by some single RFP-positive cells slightly visible at 72 h past infection. (B) Counting of RFP-positive cells (RPC) was performed+/−doxycycline (48 h post induction, 1 µg/ml). Columns in dark grey represent the number of RPCs/f.o.v., column in light grey represents the mean ratio of induction (number of RFP-positive cells in presence or absence of doxycycline). Histograms represent the calculated means+/−SD. (C) Relative intensity of red fluorescence was recorded in single cells by means of a ROI analysis using MPI-Tool imaging software, error bars signify the SD. ***, *P*<0.001 compared to non-induced cells (Mann-Whitney Rank Sum Test).

To study drug dose- and time-dependent variations of induced gene expression in cell culture, RFP expression was quantified after infection of Gli36ΔEGFR cells with HET6C-*tk39* in untreated and doxycycline-treated (0.01 µg/ml–1.0 µg/ml) cells. 24, 48 and 72 h after induction fluorescence microscopy was performed to count RFP-positive cells (RPC). The HET6C-*tk39* system demonstrated a doxycycline dose- and time-dependent increase in RFP-positive cells (RPC) (**Figure 2A**). Quantification of numbers of RFP-positive cells as well as relative red fluorescence per single cell showed a significant increase in the number of RPC (422.1±358.9 versus 1.5±2.1 RPC per field of view, mean±SD; with a ratio of induction, mean±SD: 492.6±214.4; *P*<0.001, Mann-Whitney Rank Sum Test; **Figure 2B**) and relative red fluorescence (mean±SD: 165.1±66.0 versus 25.1±12.1; mean ratio of induction±SD: 6.0±3.2; *P*<0.001, Mann-Whitney Rank Sum Test; **Figure 2C**) in doxycycline-treated versus untreated cells (1 µg/ml, 48h).

The HSV-Switch-TG17 system leads to a significant increase in a) the number of eGFP-positive cells (*P*<0.001, Mann-Whitney Rank Sum Test) in the presence of mifepristone with a ratio of induction, mean±SD of 14.0±8.8; and b) eGFP expression per single cell (ratio of induction, mean±SD: 3.6±1.9; *P*<0.01) (**see supporting information: Figure S1 A–C**). Both systems show some leakiness of the construct but improvement on tightness was especially achieved with the HET6C system, which is reflected in an increased ratio of induction.

### Imaging inducible HSV-1 amplicon vector-mediated gene expression *in vivo* by PET

**Figure 3:**
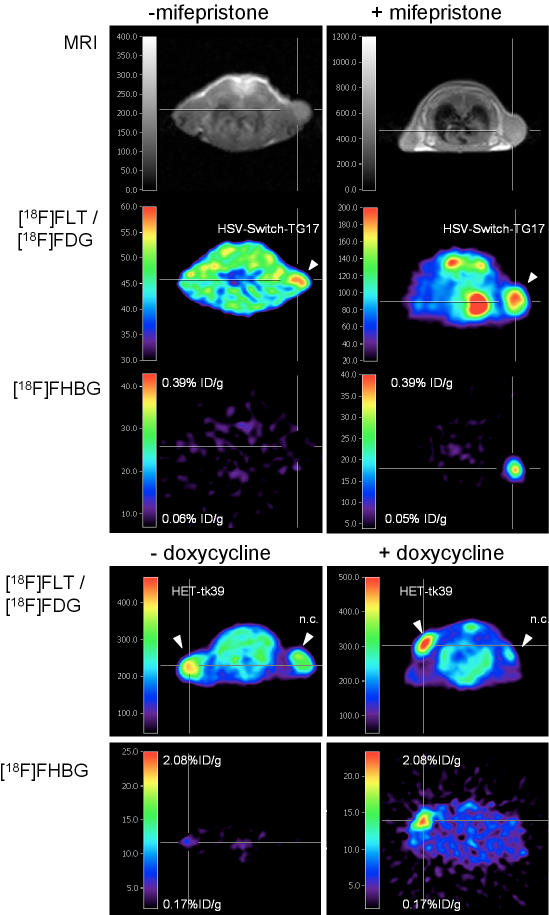
*In vivo* imaging of regulated gene expression. Multimodal imaging ([^18^F]FLT-, [^18^F]FDG- and [^18^F]FHBG-PET) of inducible gene expression by PET in nude mice bearing subcutaneous human Gli36ΔEGFR xenografts employing HSV-Switch-TG17 or HET6C-*tk39*. Mice were randomized to mifepristone- or doxycycline-treated (each group n = 7) and untreated (n_-mif_ = 8; n_-dox_ = 12) groups. Indicated xenografts (arrowhead) were injected with HSV-Switch-TG17 and HET6C-*tk39* respectively (>2×10^6^ t.u.) 48h prior to PET-imaging, resulting in high accumulation of [^18^F]FHBG in HSV-Switch-TG17 or HET6C-*tk39* infected and mifepristone- or doxycycline-treated tumors as compared to some background activity in non-treated tumors.

To investigate inducible *HSV-1-tk39* gene expression *in vivo* by PET, subcutaneous human Gli36ΔEGFR gliomas grown in nude mice were infected with HSV-Switch-TG17 or HET6C-*tk39* employing imaging-guided vector administration [6]. Animals were treated either with or without the appropriate inducer. [^18^F]FHBG-PET images were acquired 24–48 h after infection and induction demonstrating distinct accumulation of [^18^F]FHBG in both HSV-Switch-TG17 or HET6C-*tk39*-infected gliomas of mifepristone or doxycycline-treated animals, respectively, as compared to infected gliomas of untreated animals (**Figure 3**). Quantification of microPET signals for *in vivo* transduced tumors showed a 4.8-fold induction of TKGFP expression by mifepristone, with significantly higher values for mifepristone-treated animals (n = 7) versus untreated animals (n = 8) [specific accumulation 0.24±0.17 vs. 0.05±0.08%ID/g; *P*<0.05, Student's *t*-test]. Similar results were obtained in nude mice bearing subcutaneous human Gli36ΔEGFR gliomas infected with HET6C-*tk39* in the presence (n = 7) or absence (n = 12) of doxycycline [mean ratio of induction 4.7, specific accumulation 0.47±0.35 vs. 0.10±0.08%ID/g; *P*<0.01, Student's *t*-test].

### Imaging the dynamics of regulated HSV-1 amplicon vector-mediated gene expression in vivo by BLI

**Figure 4:**
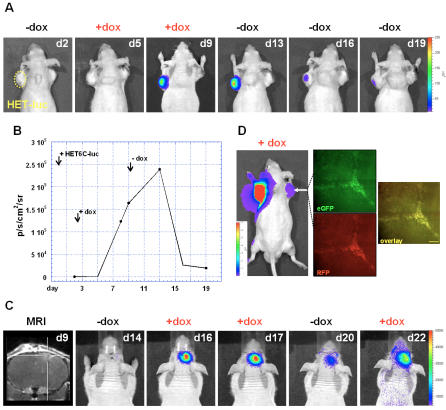
*In vivo* bioluminescence imaging of doxycycline-dependent gene expression over time. Bioluminescence imaging of induced LUC expression and image validation by histology. Unit for all color scales as well as the histogram on temporal analysis was defined as photons/second/cm^2^/steradian (p/s/cm^2^/sr). (A) Temporal analysis of up- and down-regulation of LUC expression. HET-6C injection was performed intratumorally at day 0. Days where bioluminescent images were obtained are indicated at the upper right corner, days of doxycycline treatment at the top. (B) Quantitative analysis of luciferase signal (OFF-ON-OFF) in response to doxycycline. (C) Temporal analysis of up- and down-regulation of LUC expression in the intracranial glioma model (OFF-ON-OFF-ON). Indicated are the days of tumor growth. (D) Image validation by histology. BLI of a mouse bearing a subcutaneous glioma stably expressing LUC on its left shoulder after *in vivo* transduction with HET6C-*luc* in the tumor on the right shoulder. Representative histological sections taken from the *in vivo* transduced tumor showing co-localization of eGFP, expressed constitutively from the herpes viral immediate early 4/5 promoter (Figure 1), and RFP, expressed from the bi-directional regulated promoter (Scale bar overlay: 150 µm, exposure time: 0.5 s).

To further investigate the dynamics of regulated gene expression *in vivo*, bioluminescence imaging was performed in subcutaneous and intracranial xenografts of human Gli36ΔEGFR cells. To analyze the dynamics of gene regulation within the same animals, bioluminescence signal was determined pre- and post doxycycline treatment. Furthermore, animals (n = 4) were subjected to on- and off- treatment demonstrating up- and down-regulation of *luc* gene expression *in vivo* (**Figure 4A**). Animals showed an increase in luciferase signal starting 3 days after onset of doxycycline treatment. After withdrawal of doxycycline LUC-derived signal decreased with a latency of 3 days to background values 7 days after removal of the inducer (**Figure 4B**). All animals showed a 11.8-197.5-fold increase in luciferase-derived signal after doxycycline treatment in the *in vivo* transduced tumor.

Finally, the subsequent on-, off-, on-switching of HET6C-*luc*-mediated luciferase expression was analyzed in intracranial growing human Gli36ΔEGFR gliomas in nude mice (n = 5; **Figure 4C**). At a basal state no signal was observed (d14). After doxycycline treatment (2–3 days) the signal increases up to 9.9-fold (d17; mean 5.58±2.89). After withdrawal of doxycycline, luciferase signal decreased to nearly background levels (d20). Treatment with doxycycline again induced LUC expression up to 6.0-fold (d22; mean 4.96±0.96). This demonstrates that repetitive on- and off-switching of gene expression after *in vivo* transduction of regulated genes is possible and that the dynamics of this process can be non-invasively assessed by BLI.

### Image validation

To validate *in vivo* images, histological sections were taken from representative tumor samples from HET-infected tumors, and the distribution of eGFP and RFP expression was analyzed by fluorescence microscopy. As indicated in **Figure 4D**, constitutive expression of eGFP could be observed and co-localized to the same areas where induced RFP expression was detected.

## Discussion

This study demonstrates for the first time a detailed quantitative analysis of inducible and regulated gene expression mediated by universal HSV-1 amplicon vectors after *in vivo* transduction in experimental gliomas using imaging-guided vector administration. Multi-modal *in vivo* imaging is being employed for tumor localization (MRI), assessment of viable target tissue for vector application (FDG-/FLT-PET) and assessment of the regulated tissue-dose of vector-mediated gene expression (FHBG-PET, BLI). This study demonstrates the feasibility of imaging quantitatively the *dynamics* of co-regulated genes after transduction *in vivo*. Significant specific accumulation of [^18^F]-FHBG in infected and induced tumors by microPET indicates that imaging gene regulation may be possible even in the human application by PET employing the same marker gene/marker substrate combination.

The HSV-1 amplicon vectors mediate regulatable proportional co-expression of fluorescent genes (*rfp, egfp*) with either PET (HSV-1-*tk* and *tk39*) or optical (*firefly luciferase*) imaging genes. The essential feature of these vectors is that application of a single vector mediates (i) the responsiveness to the exogenous inducer; (ii) the presence of the responsive element driving inducible gene expression; (iii) at least two inducible marker genes for analysis in cell culture and histology using fluorescence microscopy and *in vivo* using PET and optical imaging, one of which could be replaced by any other transgene of interest. Various assays were employed to analyze and quantify gene induction using fluorescence microscopy of infected cells in culture and histology, and the respective enzymatic assays *in vivo* using PET and optical imaging. The range of induction regarding the number of fluorescent positive cells varied between factors of 14 (Gene-Switch) to 492 (Tet-On). With respect to fluorescence intensity per single cell similar induction ratios were observed (3.6- to 6.0-fold). The difference between both vector systems concerning the range of induction may be attributed to the fact that the HET-vector encodes a transcriptional silencer and carries insulator elements which substantially reduce the level of basal activity in cell culture. It should be pointed out that *in vivo* some background activity was observed in the radiotracer assay for both vectors (PET) which was not the case for the bioluminescence assay. The ratio of induction as determined *in vivo* by PET did not show a difference between the vectors. As for both vectors some background activity of [^18^F]FHBG accumulation was observed *in vivo* the rate of induction of 4.7- and 4.8-fold did not reflect the range of induction as determined by induced numbers of fluorescence positive cells but by the increase of fluorescence intensity per single cell (3.6- to 6.0-fold).

The non-invasive assessment of induced gene expression *in vivo* with PET was demonstrated first by Sun et al. [31] using HeLa Tet-On cells specifically engineered to express the transactivator rtTA and stably transfected with a plasmid containing a bi-directional doxycycline responsive promoter driving expression of two PET-reporter genes. In our study, a single viral vector was engineered containing all relevant information to induce and monitor expression of the target gene *in vivo*, thus reflecting the situation which might be applied in a diagnostic or therapeutic paradigm for clinical application. The levels of TK expression as measured as %ID/g tissue were found to be within the range of values obtained with tumors infected with HSV-1 amplicon vectors expressing the viral thymidine kinase under control of a constitutive promoter [6], [34].

Regarding non-invasive assessment of inducible LUC expression, we noted that by using HET6C-*luc* the BL images showed a clear difference between non-induced and induced HET-infected tumors. The quantitative range of induction did not clearly match the one determined by PET imaging most likely due to the semi-quantitative nature of BLI data. For pre-clinical evaluation, this study demonstrates that optical imaging is a useful modality to investigate the dynamics of regulated gene expression (OFF-ON-OFF-ON) without the need for radiotracers and irrespective of decay time. Further benefits of bioluminescence imaging include minor costs, shorter scanning times, simultaneous scanning of several animals, and its relatively easy use [4]. Several types of vectors have recently been created that express optical imaging genes, e.g., firefly or Renilla and Gaussia luciferases as reporter genes to monitor gene expression *in vivo* [36]–[41] also by means of gene induction [42], [43]. However, it should be kept in mind that optical imaging does not provide fully quantitative and tomographic information which will restrict the value for clinical application [44], [45]. In contrast, PET has gained more importance in clinically approved molecular imaging technologies, especially in cancer imaging and treatment [46]. Furthermore, PET reporter genes have the advantage of giving fully quantitative tomographic information which can be directly transferred to clinical application using new generations of high resolution PET scanners [47].

Vectors combining inducible and regulatable gene expression systems and PET reporter genes will be of great interest for the further development of gene therapeutic vectors and their potential use in clinical application. By co-expressing therapeutic genes with imaging marker genes, an indirect assessment of any therapeutic gene is possible. Furthermore, the use of bi-directional promoters can be used to combine expression of two independent genes as demonstrated in this study. Recently, Dumortier et al. 2005 [43] described the use of an adenoviral vector encoding for firefly luciferase and interferons α, β and γ, respectively, co-expressed under control of the bi-directional tet-responsive promoter (P_BI-1_), thus allowing to study the effects of local cytokine expression in a hepatitis mouse model demonstrating further the potential of the bi-directional inducible promoter system.

In conclusion, universal HSV-1 amplicon vectors were generated capable of transducing inducible and regulatable gene expression in cell culture and *in vivo* which allow monitoring of the *quantitative dynamics* of regulated gene expression by means of non-invasive fluorescence, bioluminescence and PET imaging. These types of vectors will be of great interest in the further development of gene therapy for clinical application, where tight regulation of gene expression and technology for non-invasive assessment of the efficiency and safety of vectors is of critical importance.

## Materials and Methods

### Cell culture

Human Gli36ΔEGFR glioma cells (Dr. David Louis, Molecular Neurooncology Laboratory, MGH, Boston, MA) were grown as monolayers in Dulbecco's modified Eagle medium (DMEM; Life Technologies, Karlsruhe, Germany) supplemented with 10% fetal bovine serum (FBS, Roche Diagnostics, Mannheim, Germany), 1% penicillin and 1% streptomycin (P/S, Life Technologies ) at 37°C in a 5% CO_2_/95% air atmosphere.

### HSV-1 amplicon plasmid construction and helper virus-free packaging

Construction of the inducible double-gene co-expression vectors was performed by PCR-amplification and standard cloning techniques (**see supporting information: Text S1**). Helper virus-free stocks of the HSV-1 amplicon construct pHSV-Switch-TG17 and pHET-6C-*tk39* were generated as described previously [34].

### Functional assessment of gene expression in culture

To assess HSV-1 amplicon vector-mediated regulation of *tkgfp, rfp* and *tk39* gene expression, Gli36ΔEGFR cells were infected with HET6C-*tk39* or HSV-Switch-TG17 in the absence or presence of the appropriate inducer, and protein expression of HSV-1-TK, the number of RFP- or eGFP-positive cells (RPCs or GPCs) and the level of RFP- or eGFP-fluorescence were investigated as described previously [34].

### (i) Western Blot

To provide analysis of the induced expression of the *tkgfp* fusion and the *tk39* genes, human Gli36ΔEGFR glioma cells were infected with HSV-Switch-TG17 and HET6C-*tk39*, respectively, using a multiplicitiy of infection (MOI) of 3. Twenty-four hours after infection cells were lysed in buffer containing 10 mM Tris pH 8.0, 1 mM EDTA, 1 mM DTT, 0.5% Triton X-100 and protease-inhibitor-mix (Roche Diagnostics GmbH, Mannheim, Germany). The amount of protein present in each sample was quantified using a BCA Protein Assay (Pierce, Rockford, IL, USA). Equal amounts of denatured (95°C, 5 min) protein were separated using SDS-PAGE and blotted to PVDF membrane (Amersham Pharmacia Biotech, Freiburg, Germany) in standard Tris-glycine transfer buffer. After blocking of non-specific binding sites with 5% non-fat dry milk in 0.3% PBS-Tween20 (PBST) for 1 hour the membrane was washed 3 times with PBST and incubated for 1 hour with a rabbit polyclonal anti-HSV-1-TK antibody (a kind gift from Dr. Margeret Black, Washington State University, Pullmann, WA) (1:20,000 in 5% dry milk in PBST). Afterwards the membrane was washed and incubated for 1 hour with the secondary antibody (1∶20,000 in PBST; goat anti-rabbit Dianova). Proteins were visualized using the SuperSignal West Pico Chemiluminescence substrate (Pierce, Rockford, IL, USA).

### (ii) Quantification of RFP- and eGFP-positive cells (RPCs, GPCs)

For determination of RPCs or GPCs, human Gli36ΔEGFR glioma cells were plated in 24-well plates (2×10^5^ cells per well) to form confluent monolayers. Twenty four hours later, cells were infected with HET6C-*tk39* or HSV-Switch-TG17 at an MOI of 0.3. Two hours after infection, the medium was replaced by medium containing either doxycycline (dox; 0.01–1.0 µg/ml) or mifepristone (MF; 1×10^−8^–1×10^−7^M) or no inducer. Representative pictures were taken 24, 48 and 72 hr after infection. For each time point, RPCs and GPCs, respectively, were counted in at least four fields of view. To determine the ratio of induction, GPCs and RPCs were counted in the presence and absence of inducer, and the ratio (RPCs+dox/RPCs-dox or GPCs+MF/GPCs-MF) was calculated.

### Animal Experiments

All animal procedures were in accordance with the German Laws for Animal Protection and were approved by the local animal care committee and the Bezirksregierung Köln.

### (i) Tumor model and imaging-guided vector application in vivo

To study HSV-1 amplicon vector-mediated *tk* gene expression *in vivo*, subcutaneous Gli36ΔEGFR tumors, vector application and PET imaging were performed as described previously [6], [34]. Multiple sets of animals were analyzed: HET6C-*tk39* (n = 19), HSV-Switch-TG17 (n = 15) and HET6C-*luc* (n = 4), vector doses ranging between 2×10^6^ to 3×10^7^ t.u. in 50 µl of PBS. Moreover, Gli36ΔEGFR cells (1×10^5^ cells in 2 µl of DMEM) were stereotactically implanted into the right striatum of nude mice (n = 5). For application of HET6C-luc vector in pre-established i.c. gliomas, 5×10^5^ t.u. HET6C-luc were stereotactically infused over 10 min. In the case of infection with HSV-Switch-TG17, mice were given mifepristone intraperitoneally (500 µg/kg body weight dissolved in sesame oil) or sesame oil as a control, whereas mice infected with HET6C-vectors were treated with or without doxycycline (2 mg/ml) via drinking water. To determine the rate of induced gene expression *in vivo*, PET and bioluminescence imaging was performed 24–48 hours after vector administration.

### (ii) Radiosynthesis of radiotracers [^18^F]FDG, [^18^F]FLT and [^18^F]FHBG was performed as described previously [6], [34]

### (iii) PET

To avoid vector application into necrotic tumor, viable tumor tissue was visualized by means of [^18^F]FDG- or [^18^F]FLT-PET after intravenous (i.v.; tail vein) administration of no-carrier-added [^18^F]FDG or [^18^F]FLT (no-carrier-added) into experimental animals with doses ranging between 200–300 µCi/mouse. To non-invasively assess the level of induced TKGFP or TK39 expression, no-carrier-added [^18^F]FHBG was administered i.v. (300 µCi/mouse). To study tracer accumulation and wash out, PET imaging was performed as described previously [6], [34].

### (iv) Bioluminescence imaging in vivo

Analysis of luciferase gene expression was performed on an IVIS200 optical imaging system (Xenogen Corp., USA) at multiple time points after vector application (48 hours, 2–19 days). For bioluminescence detection, mice were injected intraperitoneally with D-luciferin (4 mg/animal in 200 µl PBS) and images were acquired 10 min after luciferin injection at standard camera settings for BLI with exposure time 1–5 min. Data evaluation based on a ROI analysis of BLI images to determine maximum values in photons/s/cm^2^/sr was performed by the use of Living Image Software version 2.50 (Xenogen Corp., USA). Data were background subtracted and ratios of induction [photons/s/cm^2^/sr+dox/photons/s/cm^2^/sr–dox] were determined.

### (v) MRI

To allow exact co-registration of gene expression data obtained by PET and optical imaging, T1-weighted MR images for localization of tumors were obtained on a 1.5 T Philips Gyroscan Intera or a 7 T Bruker Biospin animal scanner prior to *in vivo* transduction.

### (vi) Histology

To validate images of induced gene expression, animals were sacrificed after imaging and tumors were processed as described previously [6], [34]. For fluorescence detection, slides were examined by use of a fluorescence microscope (Zeiss Axiovert 135, Carl Zeiss, Heidenheim, Germany).

### Statistics

Descriptive statistics and regression analysis were performed with Microsoft Excel 2002 (Microsoft Corp.). Student's *t*-test as well as Mann-Whitney Rank Sum Test were performed with SigmaStat 3.0 (SPSS Inc.); statistical significance was set at the less than 5% level (*P*<0.05), statistical tests in SigmaStat are two-tailed.

## Supporting Information

Figure S1 Regulated eGFP expression in culture. (A) Fluorescence microscopy of eGFP-expressing cells with and without mifepristone-treatment of HSV-Switch-TG17 infected cells. (B) eGFP-positive cells (GPC) were counted per field of view (f.o.v.) in the absence and presence of mifepristone (−/+MF, 24h post induction). Columns in dark grey represent the number of GPCs/f.o.v., whereas the column in light grey represents the mean ratio of induction calculated as the number of GFP-positive cells with inducer divided by the number of GFP-positive cells without inducer, error bars signify the SD. ***, P<0.001 compared to non-induced cells (Mann-Whitney Rank Sum Test). (C) Quantification of relative GFP-fluorescence was determined by recording the relative intensity of green fluorescence in single cells by means of a region-of-interest (ROI) analysis using MPITool imaging software, again error bars signify the SD. **, P<0.01 compared to non-induced cells (Student's t-test).
(7.22 MB TIF)

Text S1 (0.03 MB DOC)

## References

[1] Gambhir SS, Barrio JR, Herschman HR, Phelps ME (1999). Assays for noninvasive imaging of reporter gene expression.. Nucl Med Biol.

[2] Jacobs AH, Dittmar C, Winkeler A, Garlip G, Heiss WD (2002). Molecular imaging of gliomas.. Mol Imaging.

[3] Blasberg RG (2003). In vivo molecular-genetic imaging: multi-modality nuclear and optical combinations.. Nucl Med Biol.

[4] Contag CH, Bachmann MH (2002). Advances in in vivo bioluminescence imaging of gene expression.. Annu Rev Biomed Eng.

[5] Hoehn M, Kustermann E, Blunk J, Wiedermann D, Trapp T (2002). Monitoring of implanted stem cell migration in vivo: a highly resolved in vivo magnetic resonance imaging investigation of experimental stroke in rat.. Proc Natl Acad Sci U S A.

[6] Jacobs AH, Rueger MA, Winkeler A, Li H, Vollmar S (2007). Imaging-guided gene therapy of experimental gliomas.. Cancer Res.

[7] Jacobs A, Dubrovin M, Hewett J, Sena-Esteves M, Tan CW (1999). Functional coexpression of HSV-1 thymidine kinase and green fluorescent protein: implications for noninvasive imaging of transgene expression.. Neoplasia.

[8] Tjuvajev JG, Joshi A, Callegari J, Lindsley L, Joshi R (1999). A general approach to the non-invasive imaging of transgenes using cis-linked herpes simplex virus thymidine kinase.. Neoplasia.

[9] Yu Y, Annala AJ, Barrio JR, Toyokuni T, Satyamurthy N (2000). Quantification of target gene expression by imaging reporter gene expression in living animals.. Nat Med.

[10] Yaghoubi SS, Wu L, Liang Q, Toyokuni T, Barrio JR (2001). Direct correlation between positron emission tomographic images of two reporter genes delivered by two distinct adenoviral vectors.. Gene Ther.

[11] Saito Y, Price RW, Rottenberg DA, Fox JJ, Su TL (1982). Quantitative autoradiographic mapping of herpes simplex virus encephalitis with a radiolabeled antiviral drug.. Science.

[12] Tjuvajev JG, Stockhammer G, Desai R, Uehara H, Watanabe K (1995). Imaging the expression of transfected genes in vivo.. Cancer Res.

[13] Iyer M, Salazar FB, Lewis X, Zhang L, Carey M (2004). Noninvasive imaging of enhanced prostate-specific gene expression using a two-step transcriptional amplification-based lentivirus vector.. Mol Ther.

[14] Doubrovin M, Ponomarev V, Beresten T, Balatoni J, Bornmann W (2001). Imaging transcriptional regulation of p53-dependent genes with positron emission tomography in vivo.. Proc Natl Acad Sci U S A.

[15] Green LA, Yap CS, Nguyen K, Barrio JR, Namavari M (2002). Indirect monitoring of endogenous gene expression by positron emission tomography (PET) imaging of reporter gene expression in transgenic mice.. Mol Imaging Biol.

[16] Ponomarev V, Doubrovin M, Lyddane C, Beresten T, Balatoni J (2001). Imaging TCR-dependent NFAT-mediated T-cell activation with positron emission tomography in vivo.. Neoplasia.

[17] Gossen M, Bujard H (1992). Tight control of gene expression in mammalian cells by tetracycline-responsive promoters.. Proc Natl Acad Sci U S A.

[18] Hynes NE, Kennedy N, Rahmsdorf U, Groner B (1981). Hormone-responsive expression of an endogenous proviral gene of mouse mammary tumor virus after molecular cloning and gene transfer into cultured cells.. Proc Natl Acad Sci U S A.

[19] Wurm FM, Gwinn KA, Kingston RE (1986). Inducible overproduction of the mouse c-myc protein in mammalian cells.. Proc Natl Acad Sci U S A.

[20] Christopherson KS, Mark MR, Bajaj V, Godowski PJ (1992). Ecdysteroid-dependent regulation of genes in mammalian cells by a Drosophila ecdysone receptor and chimeric transactivators.. Proc Natl Acad Sci U S A.

[21] Mayo KE, Warren R, Palmiter RD (1982). The mouse metallothionein-I gene is transcriptionally regulated by cadmium following transfection into human or mouse cells.. Cell.

[22] Gossen M, Freundlieb S, Bender G, Muller G, Hillen W (1995). Transcriptional activation by tetracyclines in mammalian cells.. Science.

[23] Baron U, Freundlieb S, Gossen M, Bujard H (1995). Co-regulation of two gene activities by tetracycline via a bidirectional promoter.. Nucleic Acids Res.

[24] Wang Y, O'Malley BW, Tsai SY, O'Malley BW (1994). A regulatory system for use in gene transfer.. Proc Natl Acad Sci U S A.

[25] Burcin MM, Schiedner G, Kochanek S, Tsai SY, O'Malley BW (1999). Adenovirus-mediated regulable target gene expression in vivo.. Proc Natl Acad Sci U S A.

[26] Furth PA, St Onge L, Boger H, Gruss P, Gossen M (1994). Temporal control of gene expression in transgenic mice by a tetracycline-responsive promoter.. Proc Natl Acad Sci U S A.

[27] Shockett PE, Schatz DG (1996). Diverse strategies for tetracycline-regulated inducible gene expression.. Proc Natl Acad Sci U S A.

[28] Kistner A, Gossen M, Zimmermann F, Jerecic J, Ullmer C (1996). Doxycycline-mediated quantitative and tissue-specific control of gene expression in transgenic mice.. Proc Natl Acad Sci U S A.

[29] Fotaki ME, Pink JR, Mous J (1997). Tetracycline-responsive gene expression in mouse brain after amplicon-mediated gene transfer.. Gene Ther.

[30] Wang Y, DeMayo FJ, Tsai SY, O'Malley BW (1997). Ligand-inducible and liver-specific target gene expression in transgenic mice.. Nat Biotechnol.

[31] Sun X, Annala AJ, Yaghoubi SS, Barrio JR, Nguyen KN (2001). Quantitative imaging of gene induction in living animals.. Gene Ther.

[32] Fraefel C, Song S, Lim F, Lang P, Yu L (1996). Helper virus-free transfer of herpes simplex virus type 1 plasmid vectors into neural cells.. J Virol.

[33] Black ME, Newcomb TG, Wilson HM, Loeb LA (1996). Creation of drug-specific herpes simplex virus type 1 thymidine kinase mutants for gene therapy.. Proc Natl Acad Sci U S A.

[34] Jacobs AH, Winkeler A, Hartung M, Slack M, Dittmar C (2003). Improved herpes simplex virus type 1 amplicon vectors for proportional coexpression of positron emission tomography marker and therapeutic genes.. Hum Gene Ther.

[35] Freundlieb S, Schirra-Muller C, Bujard H (1999). A tetracycline controlled activation/repression system with increased potential for gene transfer into mammalian cells.. J Gene Med.

[36] Lipshutz GS, Gruber CA, Cao Y, Hardy J, Contag CH (2001). In utero delivery of adeno-associated viral vectors: intraperitoneal gene transfer produces long-term expression.. Mol Ther.

[37] Bhaumik S, Gambhir SS (2002). Optical imaging of Renilla luciferase reporter gene expression in living mice.. Proc Natl Acad Sci U S A.

[38] Wu JC, Sundaresan G, Iyer M, Gambhir SS (2001). Noninvasive optical imaging of firefly luciferase reporter gene expression in skeletal muscles of living mice.. Mol Ther.

[39] Wang Y, Yu YA, Shabahang S, Wang G, Szalay AA (2002). Renilla luciferase-Aequorea GFP (Ruc-GFP) fusion protein, a novel dual reporter for real-time imaging of gene expression in cell cultures and in live animals.. Mol Genet Genomics.

[40] Tannous BA, Kim DE, Fernandez JL, Weissleder R, Breakefield XO (2005). Codon-optimized Gaussia luciferase cDNA for mammalian gene expression in culture and in vivo.. Mol Ther.

[41] Shah K, Tang Y, Breakefield X, Weissleder R (2003). Real-time imaging of TRAIL-induced apoptosis of glioma tumors in vivo.. Oncogene.

[42] Yu YA, Szalay AA (2002). A Renilla luciferase-Aequorea GFP (ruc-gfp) fusion gene construct permits real-time detection of promoter activation by exogenously administered mifepristone in vivo.. Mol Genet Genomics.

[43] Dumortier J, Schonig K, Oberwinkler H, Low R, Giese T (2005). Liver-specific expression of interferon gamma following adenoviral gene transfer controls hepatitis B virus replication in mice.. Gene Ther.

[44] Doubrovin M, Serganova I, Mayer-Kuckuk P, Ponomarev V, Blasberg RG (2004). Multimodality in vivo molecular-genetic imaging.. Bioconjug Chem.

[45] Weissleder R (2002). Scaling down imaging: molecular mapping of cancer in mice.. Nat Rev Cancer.

[46] Weissleder R (2006). Molecular imaging in cancer.. Science.

[47] Heiss W-D, Habedank B, Klein JC, Herholz K, Wienhard K (2004). Metabolic Rates in Small Brain Nuclei Determined by High-Resolution PET.. J Nucl Med.

