# Supplementary figures and images for: Switching on the Lights for Gene Therapy
Source: PLoS One. 2007 Jun 13;2(6):e528. doi: 10.1371/journal.pone.0000528 (PMC1885827; doi:10.1371/journal.pone.0000528)

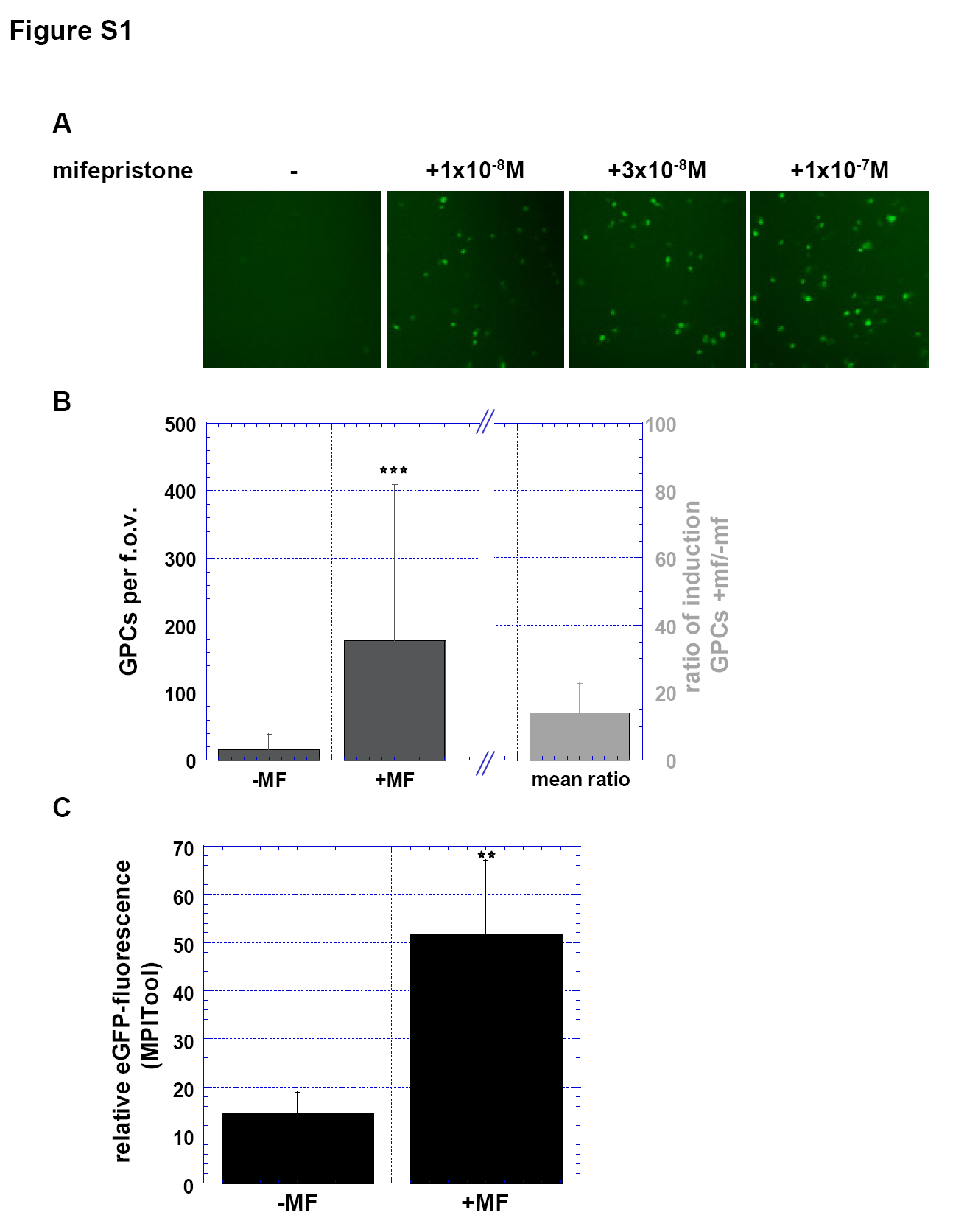

Supplement: Figure S1 — Regulated eGFP expression in culture. (A) Fluorescence microscopy of eGFP-expressing cells with and without mifepristone-treatment of HSV-Switch-TG17 infected cells. (B) eGFP-positive cells (GPC) were counted per field of view (f.o.v.) in the absence and presence of mifepristone (−/+MF, 24h post induction). Columns in dark grey represent the number of GPCs/f.o.v., whereas the column in light grey represents the mean ratio of induction calculated as the number of GFP-positive cells with inducer divided by the number of GFP-positive cells without inducer, error bars signify the SD. ***, P<0.001 compared to non-induced cells (Mann-Whitney Rank Sum Test). (C) Quantification of relative GFP-fluorescence was determined by recording the relative intensity of green fluorescence in single cells by means of a region-of-interest (ROI) analysis using MPITool imaging software, again error bars signify the SD. **, P<0.01 compared to non-induced cells (Student's t-test). (7.22 MB TIF) [file pone.0000528.s001.tif]
