# Supplementary material for: Switching on the Lights for Gene Therapy
Source: PLoS One. 2007 Jun 13;2(6):e528. doi: 10.1371/journal.pone.0000528 (PMC1885827; doi:10.1371/journal.pone.0000528)
Supplement: Text S1 — (0.03 MB DOC) [file pone.0000528.s002.doc]

**Text S1**

#### HSV-1 amplicon plasmid construction

Construction of the inducible double-gene co-expression vectors was performed by PCR-amplification and standard cloning techniques.

*Generation of pHSV-Switch-TG17*. The HSV-1 amplicon backbone pHSV-GN (kind gift of Dr. Xandra O. Breakefield, Neurogenetics Unit, MGH, Boston, MA) as well as the plasmids pSwitch (Invitrogen), pGene (Invitrogen) and pTG17 were used to generate the mifepristone-inducible HSV-1 amplicon vector HSV-Switch-TG17 (**Figure** **1**). To introduce the regulator Gal4-hPR-LBD-p65 into our HSV-1 amplicon vector system we generated a synthetic oligo, including two new restriction sites AscI and AflII, which was ligated with the 7.0kb BsrGI-BstEII fragment of pHSV-GN to form pHSV-GΔN. The gene encoding for Gal4-hPR-LBD-p65 was amplified by PCR on pSwitch (primer pair 5’-ATACTGTACACGCGCACATTTCCCCGAAAAG-3’ and 5’-GACTAGTAAGCCATAGAGCCCACCGCATC-3’; MWG Biotech, Ebersberg, Germany) and amplified material was digested with BsrGI and SpeI. The fragment was ligated into plasmid pHSV-GΔN which was previously cut with BsrGI and SpeI giving rise to plasmid pHSV-Switch. A BglII-NotI fragment of 1900bp was isolated from pTG17 and was ligated with the 4.6kb BamHI-NotI fragment of pGene to create the vector pGeneTG17. Amplification of the inducible gene Gal4-TG17 was performed by standard PCR with the following primer pair (5’-CGGCCGTTTAAACCGCTGATCAGTT-3’ and 5’-CGCGGTCGACGGTATACAGACATGA-3’) on pGeneTG17. Amplified material was digested with AflII and AscI and cloned into the AflII-AscI site of pHSV-Switch, leading to the inducible HSV-1 amplicon vector pHSV-Switch-TG17.

*Construction of pHET-6C-tk39.* To generate the HSV/EBV/TET tribrid amplicon plasmid HET6C-*tk39*, the vectors pCD-I-Tk39GFP as well as p553.1.1 and HET6C-*luc* were used. The HSV-1-*tk39* gene (kind gift of Margeret Black) was amplified by using primer pair 5’-GAA CGC GGC CGC CAC GGG GAC GTG GTT TTC CTT TG-3’ and 5’-GTG TGC GGC CGC TTC AGT TAG CCT CCC CCA TCT CCC-3’ on pCD-I-Tk39GFP. The plasmid 553.1.1-*tk39* was constructed by inserting the HSV1-*tk39*/NotI fragment into the NotI sites of the 553.1.1 plasmid. The resulting plasmid contains the *tk39* gene as well as the *rfp* gene under the transcriptional control of the bi-directional tet-responsive promoter flanked by two copies on each side of the 1.2 kb cHS4 insulator (Ins) obtained from the plasmid pJC13-1. pHET6C-*tk39* was constructed by inserting the 10kb PacI fragment of 553.1.1-*tk39* into the PacI sites of a pREHGCa-derived EBV/HSV hybrid amplicon containing a bidirectional expression cassette for a Tet-responsive reverse transactivator (rtTA) and silencer (tTS), and a CMV-driven expression cassette for tTS.
